# A tool for evaluating heterogeneity in avidity of polyclonal antibodies

**DOI:** 10.3389/fimmu.2023.1049673

**Published:** 2023-02-16

**Authors:** Kan Li, Michael Dodds, Rachel L. Spreng, Milite Abraha, Richard H. C. Huntwork, Lindsay C. Dahora, Tinashe Nyanhete, Sheetij Dutta, Ulrike Wille-Reece, Erik Jongert, Katie J. Ewer, Adrian V. S. Hill, Celina Jin, Jennifer Hill, Andrew J. Pollard, S. Munir Alam, Georgia D. Tomaras, S. Moses Dennison

**Affiliations:** ^1^ Center for Human Systems Immunology, Duke University, Durham, NC, United States; ^2^ Department of Surgery, Duke University, Durham, NC, United States; ^3^ Integrated Drug Development, Certara, Seattle, WA, United States; ^4^ Duke Human Vaccine Institute, Duke University, Durham, NC, United States; ^5^ Department of Immunology, Duke University, Durham, NC, United States; ^6^ Structural Vaccinology Lab, Malaria Biologics Branch, Walter Reed Army Institute of Research, Silver Spring, MD, United States; ^7^ PATH's Center for Vaccine Innovation and Access, Washington, DC, United States; ^8^ GSK Vaccines, Rixensart, Belgium; ^9^ The Jenner Institute, University of Oxford, Oxford, United Kingdom; ^10^ National Institute for Health and Care Research (NIHR) Oxford Biomedical Research Center, Oxford, United Kingdom; ^11^ Oxford Vaccine Group and Department of Pediatrics, University of Oxford, Oxford, United Kingdom; ^12^ Department of Pathology, Duke University, Durham, NC, United States; ^13^ Department of Molecular Genetics and Microbiology, Duke University, Durham, NC, United States

**Keywords:** dissociation rate, avidity, binning, polyclonal antibodies, Typhim, RTS,S/AS01, biolayer interferometry (BLI), Typbar TCV

## Abstract

Diversity in specificity of polyclonal antibody (pAb) responses is extensively investigated in vaccine efficacy or immunological evaluations, but the heterogeneity in antibody avidity is rarely probed as convenient tools are lacking. Here we have developed a polyclonal antibodies avidity resolution tool (PAART) for use with label-free techniques, such as surface plasmon resonance and biolayer interferometry, that can monitor pAb-antigen interactions in real time to measure dissociation rate constant (*k_d_*) for defining avidity. PAART utilizes a sum of exponentials model to fit the dissociation time-courses of pAb-antigens interactions and resolve multiple *k_d_* contributing to the overall dissociation. Each *k_d_* value of pAb dissociation resolved by PAART corresponds to a group of antibodies with similar avidity. PAART is designed to identify the minimum number of exponentials required to explain the dissociation course and guards against overfitting of data by parsimony selection of best model using Akaike information criterion. Validation of PAART was performed using binary mixtures of monoclonal antibodies of same specificity but differing in *k_d_* of the interaction with their epitope. We applied PAART to examine the heterogeneity in avidities of pAb from malaria and typhoid vaccinees, and individuals living with HIV-1 that naturally control the viral load. In many cases, two to three *k_d_* were dissected indicating the heterogeneity of pAb avidities. We showcase examples of affinity maturation of vaccine induced pAb responses at component level and enhanced resolution of heterogeneity in avidity when antigen-binding fragments (Fab) are used instead of polyclonal IgG antibodies. The utility of PAART can be manifold in examining circulating pAb characteristics and could inform vaccine strategies aimed to guide the host humoral immune response.

## Introduction

Avidity of polyclonal antibodies (pAbs) in serum, plasma and mucosal fluids refers to the overall strength of pAbs-antigen binding and depends on the affinities of pAbs for the antigen and the valency of pAbs and the antigen. Avidity of pAbs for a given antigen can be related to their functional efficiency (1–4). Avidity measurement is important to monitor affinity maturation of the humoral response to vaccines and can aid in developing immunization strategies, such as using different engineered immunogens, adjuvants and routes, to guide pAb affinity maturation against desired protective epitopes (5). PAb avidity data is also used to measure the incidence of recent infections as low avidity antibodies are mounted after the onset of infection (6, 7). Moreover, avidity measurements are immensely helpful in immune correlate analysis of vaccines as higher avidity antibodies may associate with protection (3, 8, 9).

While avidity is classically measured by monitoring binding under chaotropic conditions using ELISA and other methods (10), kinetics based methods for measuring avidity of pAbs using surface plasmon resonance (SPR) and biolayer interferometry (BLI) techniques have been widely employed (8, 11–22). Both SPR and BLI are label-free techniques for studying kinetics of biomolecular interactions where one of the binding partners is immobilized (ligand) on an appropriate sensor chip or surface and the other binding partner, termed as analyte, is kept in solution to monitor interaction. Specific binding time-courses are obtained by appropriate reference subtraction to remove binding responses due to non-specific interactions. The association and dissociation rate constants (*k_a_* and *k_d_* respectively) and the apparent dissociation constant (*K_D_*) of the interaction are then obtained by fitting the specific binding time-courses globally to a 1:1 binding model. The *K_D_* values for monoclonal antibodies (mAbs) interacting with immobilized ligands determined using a 1:1 binding model will include the avidity effect due to the bivalency of antibodies resulting in slower *k_d_* unless the ligands are immobilized at surface densities low enough to remove avidity effect. When pAbs are studied, the concentrations of the interacting antibodies at clonal level remain unknown and so the *k_a_* and hence the *K_D_* cannot be determined. However, the *k_d_*, which is concentration independent, can be readily measured. Since the *k_d_* is inversely related to the stability of antigen-antibody complex, the avidity of the pAbs for a given antigen can be inferred by simply measuring the *k_d_* of the interaction. A slower (smaller) *k_d_* value measured would indicate higher avidity of pAbs and a faster (larger) *k_d_* value estimated would indicate lower avidity of the pAbs. The label-free real-time detection of interactions by SPR and BLI techniques enables monitoring the dissociation of antigen-antibody complexes without the use of any chaotrope. This is particularly helpful when studying chaotrope sensitive paratope or epitope (23).

PAbs-antigen binding, unlike that of mAbs, is multifaceted because pAbs are heterogeneous and recognize a range of epitopes (24). It includes antibodies competing for certain epitope(s), parallel binding to different epitopes and the binding or displacement of antibodies of certain specificity allosterically regulated by the binding of antibodies of a different specificity. All the above manifest often in complex time-course profiles. Currently, the standard practice in SPR and BLI analysis of pAbs binding to a given antigen is to measure the antigen specific binding response as a direct readout and estimate the *k_d_* of the interaction using a Langmuir dissociation model assuming a 1:1 interaction (8, 11–22),


(1) $$R\left(t\right)=\alpha e^{-k_{\text{d}}t}$$

where *R*(*t*) is the dissociation time-course, α is the response at the beginning of dissociation and *k_d_* is the dissociation rate constant (dissociation rate from here on). Fitting of non-monophasic pAbs dissociation curves to a Langmuir dissociation model (equation 1) yields a *k_d_* that is more weighted towards antibodies with slower dissociation rates among the multiple antibodies that simultaneously interact with the antigen potentially targeting different epitopes within the antigen. The inadequacy of the Langmuir model to describe the data will be conspicuous in the residuals plot when the goodness of fit of pAbs dissociation time-courses is judged. Yet the pAbs dissociation time-courses are continued to be analyzed using Langmuir dissociation model (8, 11–22) or dissected into fast and slow phases manually (25) as convenient tools are lacking. Thus, models that account for the contribution of various antibodies differing in *k_d_* to the observed dissociation time-courses of pAbs-antigen interaction would be appropriate for better understanding of the heterogeneity in avidity of pAbs. For this purpose, we developed the **p**olyclonal **a**ntibodies **a**vidity **r**esolution **t**ool (PAART), that uses a sum of exponentials model (described in methods), for fitting the polyclonal antibody dissociation kinetics to determine the minimal number of antibody components with different *k_d_* and the respective fractions required to adequately describe the data. Our aim is to use PAART for dissociation rate (and hence the avidity) binning of the time-courses of pAbs interacting with (a) single-epitope antigens to find different bins of antibodies with varying avidity that compete for the same epitope and (b) multi-epitope antigens for understanding the overall heterogeneity in avidity as epitope specificity of PAART derived antibody components (*k_d_* values) cannot be assigned.

In this report, we first describe the validity of PAART using a simple mimic of pAbs created in a controlled fashion such as binary and ternary mixtures of mAbs targeting the same epitope with comparable *k_a_* but differing in their *k_d_*. The estimates of *k_d_* and their fractions obtained from the dissociation phase data alone of binary mixtures by PAART were comparable to the estimates predicted by a competing reactions model using both the association and dissociation phases. We then demonstrate the utility of PAART in (1) dissecting avidity heterogeneity of phase 2 clinical studies’ post-vaccination serum IgG antibodies of (a) malaria vaccinees against a single-epitope peptide antigen and (b) typhoid vaccinees against a multi-epitope polysaccharide antigen, (2) component level affinity maturation of a malaria vaccine induced serum antibody responses against different antigens, (3) comparing the avidity heterogeneity between two typhoid vaccine regimens and between protected and not-protected malaria vaccinees, and (4) enhancing refinement of avidity heterogeneity by using polyclonal antigen-binding fragments (Fab) instead of polyclonal IgG antibodies of Human Immunodeficiency Virus type-1 (HIV-1) controllers sera. Insights on avidity diverseness of vaccine induced pAbs that can be obtained from PAART analysis as illustrated here will be valuable for the characterization of the optimal antibody response of an efficacious vaccine.

## Materials and equipment

### Antigens

An amino terminal biotin-Aminohexanoic acid (biotin-Ahx) tagged peptides corresponding to the *Plasmodium falciparum* Circumsporozoite protein (PfCSP) repeat region (NPNA3; biotin-Ahx-NPNANPNANPNA with an amidated carboxy terminal, NANP6 (biotin-Ahx-NANPNANPNANPNANPNANPNANP), N-terminal junctional region (N-interface; biotin-Ahx-KQPADGNPDPNANPN with an amidated carboxy terminal) and the negative control peptide C1 (Biotin-KKMQEDVISL WDQSLKPCVK LTPLCV) were custom made by CPC Scientific (Sunnyvale, CA). A recombinant CSP (CSP) containing the N-terminal region, 3 NVDP and 19 NANP repeats followed by the C-terminal region was produced and purified as described previously (26). A World Health Organization (WHO) international standard Vi polysaccharide (Vi-PS) from C. *freundii* was obtained from the National Institute for Biological Standards and Controls, United Kingdom. A recombinant HIV-1 glycoprotein construct BG505gp140 T332N SOSIP.664 was produced as previously described (27).

### Monoclonal antibodies

The CSP central repeat region specific mAbs AB334, AB315 and AB395 were derived from individuals that participated in the RTS,S/AS01 malaria vaccine study described in Regules et al. (28). The protocol of RTS,S vaccinees plasmablasts isolation, cloning and antibodies sequencing were described in the earlier report (28). The Fab of AB334 was generated by digesting AB334 IgG1 using Fab preparation kit from Thermo Fisher Scientific (Waltham, MA) following supplied procedure.

### Study samples

Samples from participants in phase 2 clinical trials of malaria vaccines (Clinical Trial Registration: NCT01883609 and NCT01366534), typhoid vaccines (Clinicaltrials.gov ID: NCT02324751) and a HIV-1 virus controller cohort enrolled through Infectious Diseases Clinic at Duke University Medical Center were collected following informed consent. Sample analyses were performed with approval from the Duke Medicine Institutional Review Board for Clinical Investigations (Protocol Pro00074497, Pro00104803 and Pro00009701). The efficacy and/or immunological evaluations for these studies were reported earlier (8, 11, 13, 22, 29–31). All study participants had previously provided consent for future use of samples for research, and all samples were de-identified. Polyclonal IgG antibodies were purified from sera or plasma samples using Protein G HP MultiTrap plates (GE Healthcare, USA) using manufacturer provided procedure.

### Biolayer Interferometry equipment

All BLI data were collected using Fortebio OctetRed 384 instruments and biosensors (Fortebio- currently Sartorius, Fremont, CA). Both data acquisition and analyses were performed with United States Food and Drug Administration’s Title 21 Code of Federal Regulations Part 11 (FDA Title 21 CFR Part 11) compliant software versions (Data Acquisition 9.0 and Data Analysis 9.0 or 10.0 packages).

## Method

### Biolayer interferometry assay

**Figure 1:**
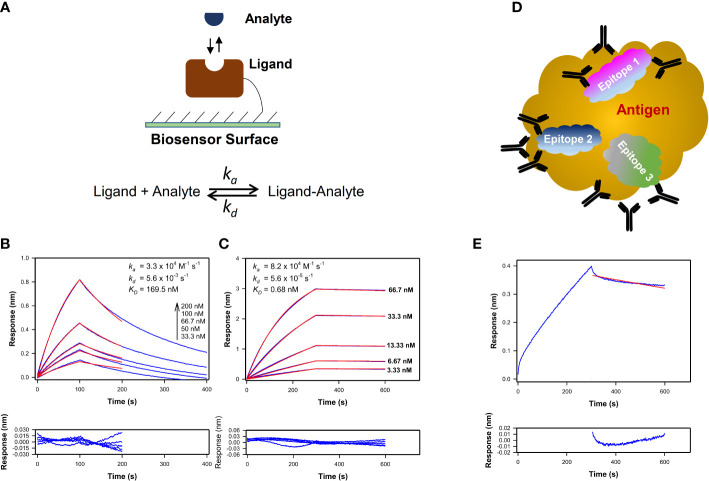
Kinetics profiles of monoclonal and polyclonal antibodies interaction with antigens. **(A)** Schematics showing the assay configuration for kinetics measurements of a homogenous analyte binding to a ligand immobilized on SPR or BLI biosensor to estimate the association and dissociation rate constants (k_a_ and k_d_ respectively) and K_D_ value. **(B, C)** Time-courses of Plasmodium falciparum circumsporozoite protein central repeat region specific mAb AB334 in its Fab form **(B)** and IgG1 form **(C)** binding (blue lines) at different indicated concentrations to a NANP repeat peptide NPNA3 with their best fit to a 1:1 binding model overlaid (red lines) on them are shown. The residuals plots of the fit are shown below the binding time-courses. The K_D_ values estimated correspond to affinity in case of AB334 Fab binding **(B)** and avidity in case of AB334 IgG1 **(C)** respectively. **(D)** Schematic of a macromolecular antigen is shown as a gold colored cloud shape. The antigen presents multiple epitopes on its surface that are targeted by polyclonal antibodies. Three such epitopes are represented as cloud shapes in different colors (Epitopes 1-3). The observed macromolecular antigen binding time-courses arise from antibodies binding to different epitopes in parallel and some antibodies competing against each other for certain epitope(s). For simplicity, only monomeric antibodies are portrayed. **(E)** As an example of polyclonal antibodies binding, 1:50 diluted serum of a malaria vaccinee in a phase 2a trial (NCT01857869) binding to the NPNA3 peptide is shown (blue line) along with the 1:1 model fit of the dissociation phase (red line). The residuals plot of the fit is shown below the sensorgram plot.

The kinetics of the biomolecular interactions were examined by immobilizing the ligand on an appropriate sensor surface and keeping the analyte in solution (**Figure 1A**). Antigens were loaded onto Streptavidin (SA) or Aminopropylsilane (APS) or Amine reactive (AR2G) biosensors as detailed previously for testing the binding antibodies (8, 11–13, 22). For this method, the form of the antibody will influence the kinetics of binding to the immobilized antigen as demonstrated by differences in the kinetics of epitope-matched Fab and IgG1 (Fab form, **Figure 1B**; IgG1 form, **Figure 1C**). The estimated *K_D_* corresponds to affinity for Fab binding (**Figure 1B**) and avidity for IgG1 (**Figure 1C**) to the antigen. Additionally, the form of the antigen will influence how many Fab-epitope interactions can occur simultaneously (**Figure 1D**). When sera or plasma that contains a polyclonal mix of antibodies (**Figure 1E**) is tested, the sensorgram plot visualizes the results from multiple antibodies binding to an antigen.

The binding of AB334 Fab, AB334, AB315, AB395 mAbs and mAbs-mixtures to NPNA3 peptide was carried out using SA sensors. The NPNA3 peptide and negative control peptide C1 (for subtracting out responses due to non-specific interactions) were loaded onto SA sensors with a loading threshold set to not exceed 0.1 nm. Baseline step was monitored by dipping NPNA3 and C1 sensors in 1x kinetics buffer wells (Fortebio- currently Sartorius, Fremont, CA), followed by association step by dipping sensors into wells containing antibodies diluted in 1x kinetics buffer. The dissociation was monitored by dipping sensors back into 1x kinetics buffer wells used in baseline step to facilitate inter-step correction. Specific binding responses were obtained by parallel referencing of C1 sensors and fitted globally to a 1:1 Langmuir binding model. The dissection of component antibody binding time-courses of binary mAbs mixtures binding to NPNA3 was performed using a heterogeneous analyte (competing reactions) model after exporting the reference subtracted time-courses to BiaEval 4.1 software (GE Healthcare Biacore LifeSciences). In the case of polyclonal samples, the standard analyses of dissociation phases were performed as per manufacturer’s technical note for dissociation rate ranking of crude samples as described earlier (8, 12, 13).

### Polyclonal antibodies avidity resolution tool (PAART)

The PAART method was developed and implemented using R statistical software version 3.6.1 (R Foundation for Statistical Computing, Vienna, Austria). Briefly, for multiple antibodies interaction with an antigen, the observed response at the beginning of the dissociation time-course should equal to the sum of binding responses from each group of antibodies with similar dissociation features (antibody component) that is bound to the antigen at the end of the association phase. Then the dissociation time course can be modeled to a sum of exponentials as below,


(2) $$R\left(t\right)=\sum_{i=1}^{n}\alpha_{i}e^{-k_{d_{i}}t}$$

where *α*_*i*_ is the response associated with antibody component *i* at time *t*=0 (beginning of dissociation phase), *k _di_* is the dissociation rate associated with antibody component *i*, *n* is the number of exponentials, and *R*(*t*) is the total response at time *t*. For each polyclonal sample, the dissociation phase is fit sequentially to the sum of exponentials model (Equation 2) for increasing values of *n* continuing until the model with *n*+1 components has a larger value of the Akaike information criterion (AIC) than the model with *n* components. AIC balances goodness of fit with complexity, guarding against overfitting of data by penalizing overly complex models. Thus, PAART analysis yields the minimal number of antibody components with different *k_d_* and the respective fractions (*f* defined in Equation 3) required to adequately describe the pAbs dissociation phase data.


(3) $$f=\;\frac{\alpha_{i}}{\sum_{i=1}^{n}\alpha_{i}}$$

The standard error of the estimates of *k*_*d*_i__, plots of time-courses calculated from the best fit overlaid on the experimental time-courses and the fitting residuals are used to judge the goodness of fit.

**Figure 2:**
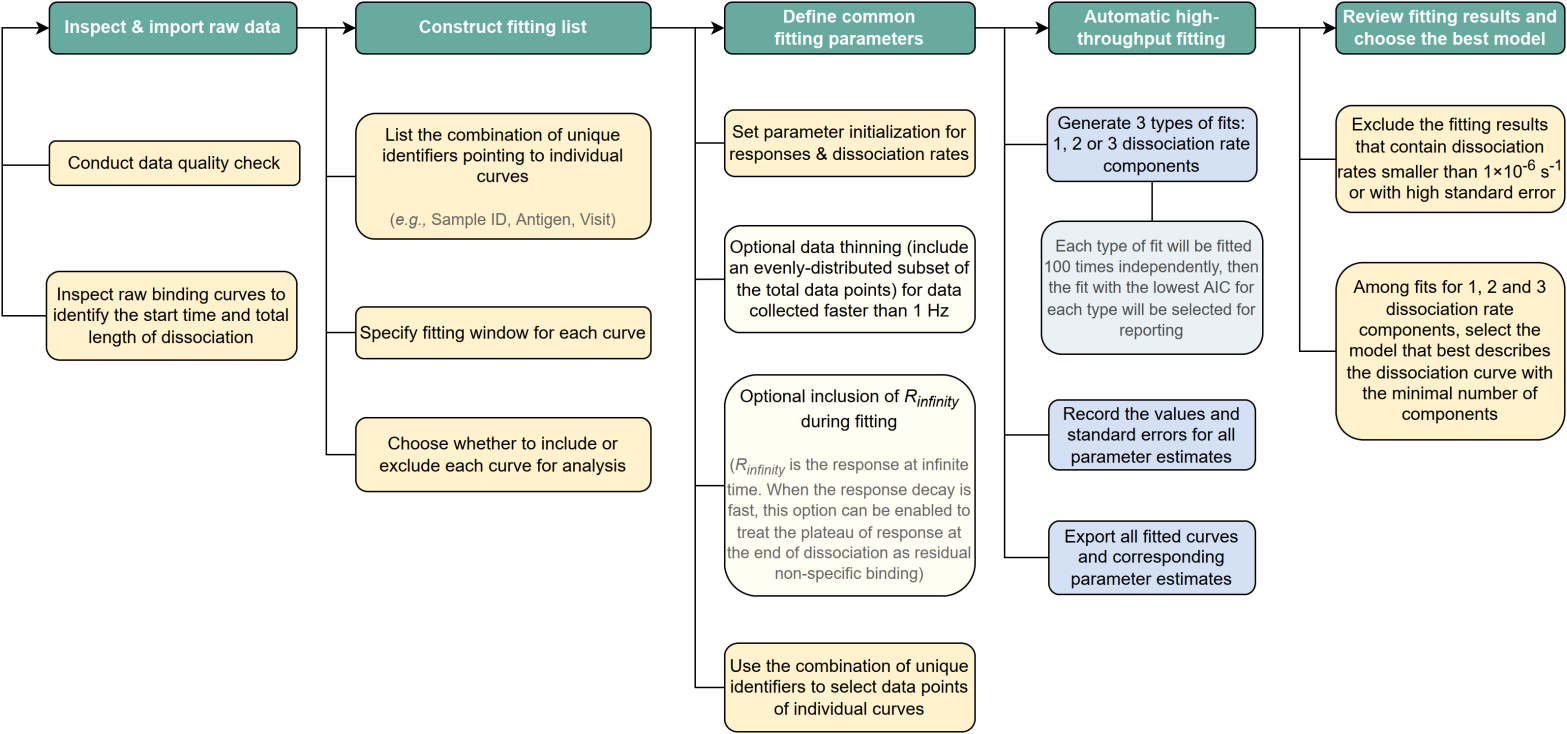
Schematic of the steps used to perform PAART analysis. A flow chart showing step-wise procedure for performing PAART analysis.

A flow chart detailing the steps used to perform PAART analysis is shown in **Figure 2**. The code for sequential fitting of dissociation phase of antibody-antigen interaction using R software along with relevant annotations and a working example can be found in GitHub^1^.

## Results

### Verification of PAART

**Figure 3:**
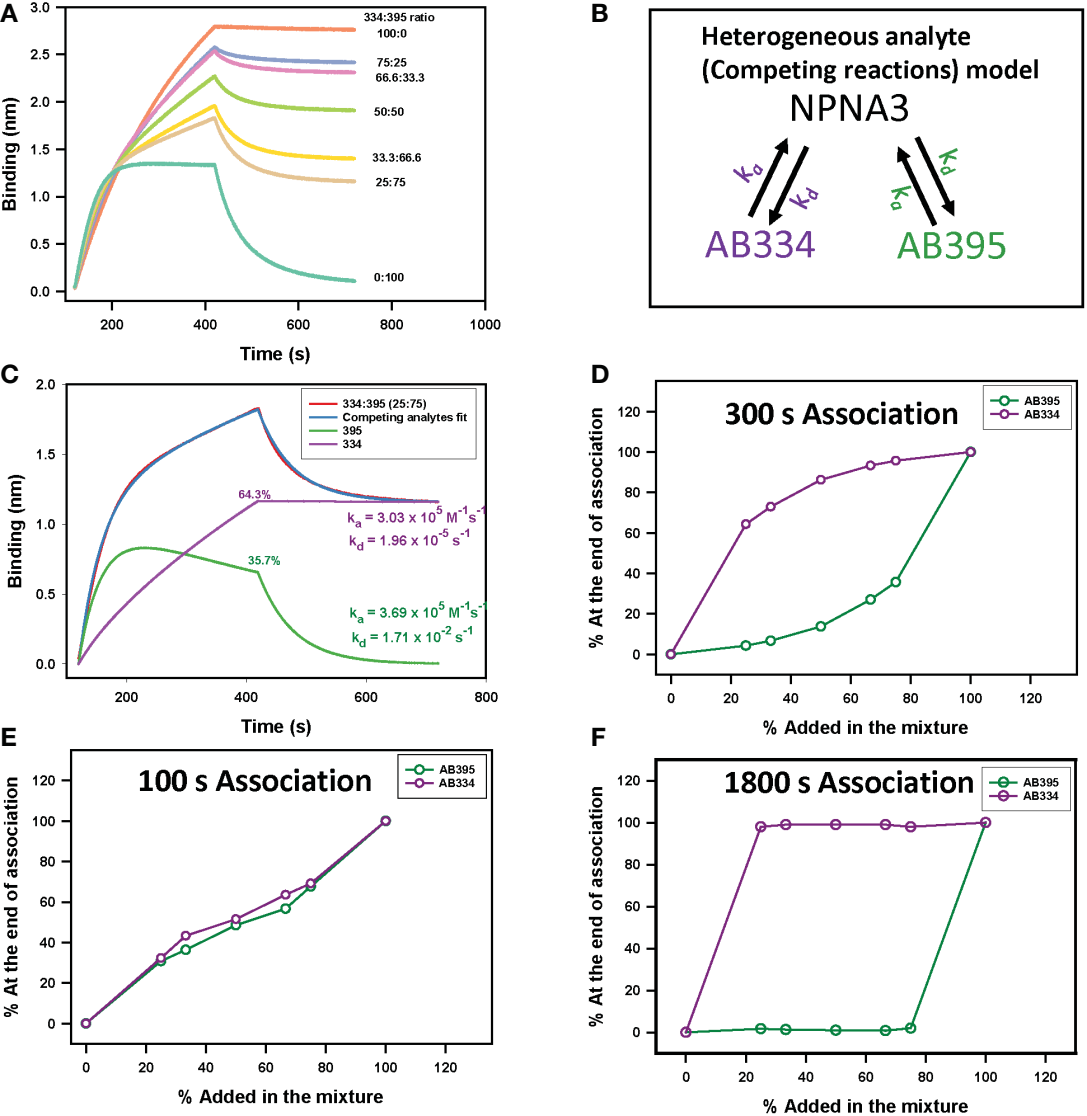
Epitope occupancy dynamics in binary antibody mixtures. **(A)** NPNA3 peptide binding time-courses of 5 µg/ml of a high avidity antibody AB334, a low avidity antibody AB395, and these two mAbs mixed at different ratios maintaining a total concentration of 5 µg/ml are shown. **(B)** Schematic of a heterogeneous analyte (competing reactions) model used to dissect total binding into component antibody binding kinetics is shown. The mAbs AB334 (purple) and AB395 (green) compete for binding to the immobilized antigen NPNA3. The *k_a_* and *k_d_* of each mAb are color coded to match the respective mAb. **(C)** Resolution of component antibodies binding time-courses contributing to the total observed binding of AB334 and AB395 mixed at 25:75 ratio predicted by a competing analytes model fit. **(D–F)** Plots of fraction of response of antibodies bound at the end of association phase **(D)** 300 s, **(E)** 100 s and **(F)** 1800 s respectively) for different compositions of the binary mixture are shown.

In order to verify whether PAART would resolve dissociation rates of different antibodies present in a polyclonal mixture, as a testing ground, we used binary mixtures of mAbs targeting the central repeat region of *Pf*CSP that consists of major repeats of NANP motif along with interspersed minor repeats of NVDP motif. The repeats region specific mAbs we used in this study differ mostly in the *k_d_* of their interaction with a synthetic peptide NPNA3 (32) that contains 2.5 NANP repeats (**Table 1**). We selected mAbs that differed in *k_d_* by 660.7-fold; a high avidity (slower *k_d_*) mAb (AB334, **Figure 1C**) and a low avidity (faster *k_d_*) mAb (AB395, **Figure S1B**) to make binary mixtures. The binding time-courses to NPNA3 of mAbs AB334 and AB395 alone and the binary mixtures of these two mAbs at various compositions are shown in **Figure 3A**. The biphasic nature of both the association and dissociation phases is evident in all binary mixtures (**Figure 3A**). We used a heterogeneous analyte (competing reactions) model (**Figure 3B**), that describes the interaction of two analytes that compete for the same ligand with different kinetics features (*k_a_* and *k_d_*), to simultaneously fit both the association and dissociation phases of the binary mixtures to obtain *k_a_* and *k_d_* pairs corresponding to the two antibodies. This analysis resolved the contribution of each competing antibody to the observed binding time-courses. A representative data is shown in **Figure 3C** for the binary mixture of AB334 and AB395 at 25:75 molar ratio. The component antibodies binding curves obtained using heterogeneous analyte model fitting of binary mixture binding curve (**Figure 3C**) reveal the dynamics of antibodies interacting with the epitope NPNA3 as follows. The low avidity mAb AB395 with a faster *k_a_* dominates the very early phase of the binding but gets replaced progressively by the high avidity mAb AB334 such that at the end of the association phase about 36% of AB395 ( $\frac{Response\;of\;AB395\;at\;the\;end\;of\;association}{Response\;of\;Binary\;mixture\;at\;the\;end\;of\;association}\times 100$ ) remained bound to NPNA3 despite being at a higher proportion (75%) in the mixture. The antibody dynamics observed in different compositions of the binary mixtures is summarized in **Figure 3D**, showing the correlation between the percentage of bound antibodies at the end of a 300 seconds association phase and the percentage of antibodies present in the binary mixtures. At equilibrium, the ratio of occupancy for the interacting epitope between the two binary antibody mixture components is simply the ratio between their *K_D_* values and their concentrations. Before attaining equilibrium, the epitope occupancy of antibodies would vary with time depending upon the kinetics features of the antibodies’ interaction with the epitope. This is evident from the component time-courses of competing antibodies in **Figure 3C** and simulations (**Figure S2**) that the length of association phase will determine the fraction of antigen occupancy of competing antibodies at the beginning of dissociation. Thus, when the association phase was shortened to 100 seconds, a higher proportion of low avidity antibody AB395 remained bound to the antigen (**Figure 3E**), whereas prolonging the association phase to 1800 seconds resulted in low proportions of AB395 remaining bound to antigen (**Figure 3F**).

**Table 1:** Characteristics of NPNA3 peptide binding of mAbs chosen for making binary and ternary mixtures.

Antibody	*k_a_* (M^-1^ s^-1^)	*k_d_* (s^-1^)	*K_D_* (nM)
AB334	8.2 × 10^4^	5.6 × 10^-5^	0.68
AB315	1.8 × 10^5^	2.4 × 10^-4^	1.36
AB395	4.7 × 10^5^	3.7 × 10^-2^	79.1

**Figure 4:**
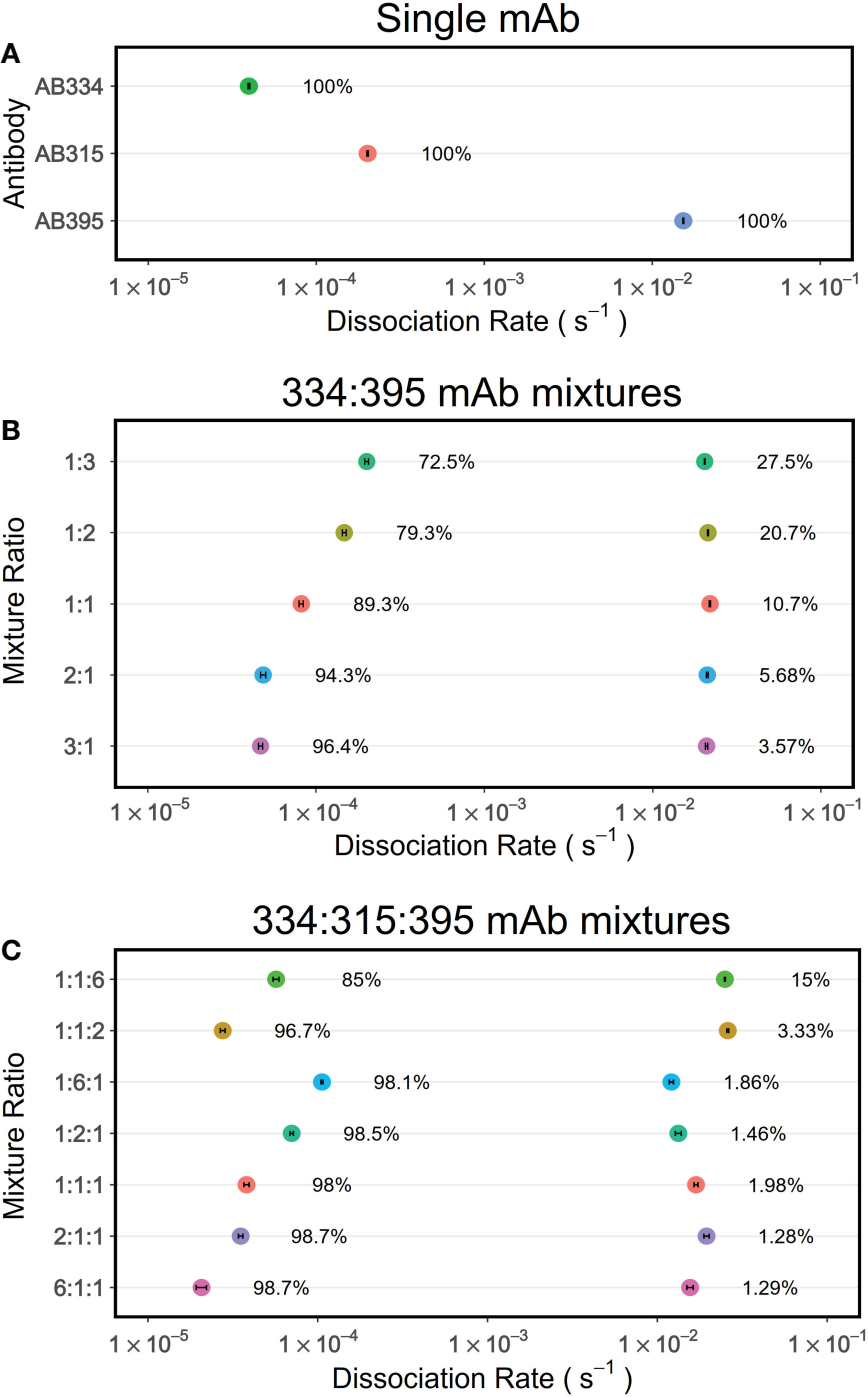
PAART analyses of dissociation phases of mAbs mixtures interacting with NPNA3 dissect different dissociation rates. The dissociation rates and their fractions estimated by PAART analysis of dissociation phases of mAbs alone **(A)**, the binary mixtures of AB334 and AB395 at various compositions **(B)** and the ternary mixtures of AB334, AB315 and AB395 **(C)** at different compositions are shown. The dissociation phases used here were recorded after 5 minutes of association phase. In each panel, the error bars associated with symbols indicate the standard error in the estimate of *k_d_* values.

The analyses above of fitting both the association and dissociation phases can be performed only when the concentrations of the antibodies in the binary mixtures are known. But when examining polyclonal samples, relevant concentrations of antibodies remain unknown, restricting the kinetics analysis to only the dissociation phase. Therefore, it is important to understand whether the sum of exponentials analysis of dissociation phases alone would recapitulate the dissected *k_d_* (from the simultaneous fits of association and dissociation phases) and their fractions appropriately. Thus, we performed PAART analysis of dissociation phases of the mAbs AB334, AB395 and their binary mixtures to obtain *k_d_* and their fractions (**Figures 4A, B**). Expectedly, two *k_d_* values were resolved in the binary mixtures with fractions similar to those obtained by fitting both the association and dissociation phases simultaneously to a competing reactions model (**Figure 3D**). We further increased the complexity by adding to the mixture a third mAb AB315 (**Figure S1A**) which has a ~4.3 fold faster *k_d_* compared to the high avidity mAb AB334 (**Table 1**). Analysis of the ternary mixtures of mAbs AB334, AB315 and AB395 dissociation phases resolved only two *k_d_* values (**Figure 4C**); one corresponding to low avidity (1-3 × 10^-2^ s^-1^) and another to high avidity (1 × 10^-5^ to 1 × 10^-4^ s^-1^). The faster *k_d_* can be assigned to the low avidity mAb AB395 whereas the slower *k_d_* appears to be an averaged *k_d_* value of the two high avidity mAbs AB334 and AB315. This lack of fine resolution in *k_d_* between the two high avidity antibody components could be due to small difference (4.3 fold) between their *k_d_*. In fact, when the binary mixtures of AB334 and AB315 were tested PAART resolved a major contributor (>96%) to the total binding with *k_d_* values ranging from 5.1 × 10^-5^ to 1.2 × 10^-4^ s^-1^ and a negligible contributor (≤3.3%) to total binding with *k_d_* of ~1× 10^-2^ s^-1^ (**Figure S3**). It is also interesting to note from **Figure 4C** that the low avidity mAb AB395 in the ternary mixture exhibited epitope occupancy of only 15% (for AB334:AB315:AB395 at 1:1:6 ratio) to <5% (for all other mixing ratios used) at the end of 300 s association phase. Taken together these results show that the PAART analysis of dissociation phases of binary and ternary mixtures of mAbs of different avidity (i.e differing in *k_d_* values) can successfully dissect low avidity antibodies (*k_d_* values ~ 1×10^-2^ s^-1^) from high avidity antibodies (*k_d_* values 1×10^-4^ - 1×10^-5^ s^-1^).

### PAART dissects avidity heterogeneity of malaria vaccine-induced polyclonal serum IgG antibodies

**Figure 5:**
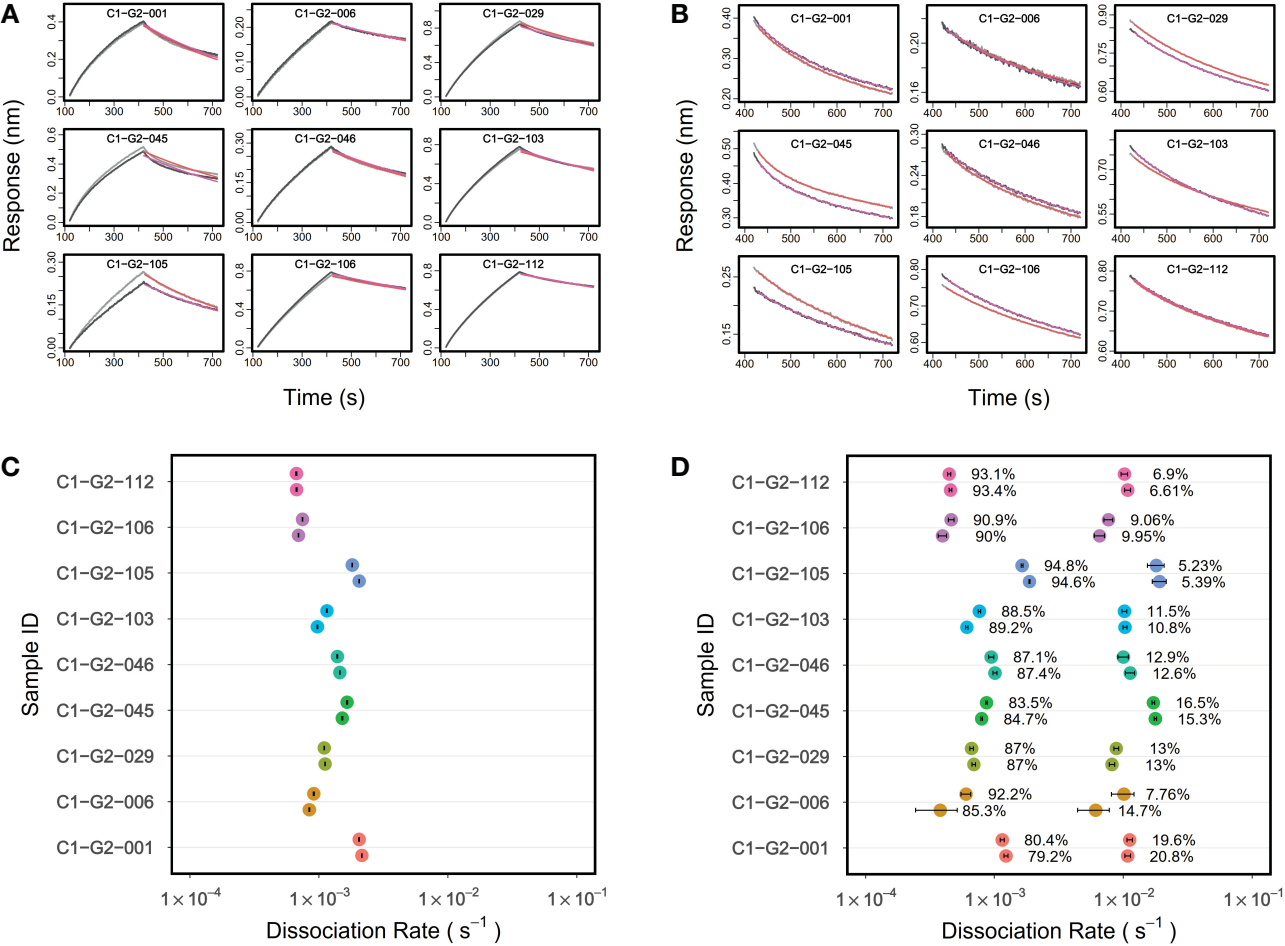
PAART analysis dissects two dissociation rates from malaria vaccinees’ serum IgG antibodies-NPNA3 interaction. **(A)** Association and dissociation time-courses in duplicate (grey and black lines) of serum IgG antibodies (at 50 µg/ml concentration) of vaccinees from a Phase 2a malaria vaccine trial interacting with NPNA3 peptide are shown along with the overlaid fits (red lines) obtained using Langmuir dissociation. The vaccinees received three doses of RTS,S/AS01 vaccine at months 0,1 and 2. IgG antibodies were purified from the post third immunization sera a day before the Pf sporozoite challenge. **(C)** The dissociation rates (in duplicate) estimated using standard Langmuir dissociation fit are displayed for different vaccinees **(B)** Dissociation time-courses of vaccinees shown in panel A with overlaid best fits from PAART analysis are shown. **(D)** PAART analysis derived dissociation rates (in duplicate) and their fractions of different vaccinees are displayed. In panels **(C, D)**, the error bars associated with symbols indicate the standard error in the estimate of *k_d_* values.

Next we used PAART to analyze the dissociation phases of vaccine induced pAbs interacting with antigens to investigate whether PAART would dissect different *k_d_* and thus help quantify the heterogeneity in avidity. For this, we used IgG antibodies purified from the sera of select vaccinees that participated in a phase 2a malaria vaccine clinical trial (Clinical Trial Registration: NCT01883609) and received RTS,S/AS01 vaccine at months 0, 1 and 2 (31). The kinetics of vaccinees’ serum IgG antibodies binding to a minimal repeat peptide NPNA3 (32) corresponding to the central repeat region of PfCSP was tested. The post-3^rd^ vaccination serum IgG antibodies drawn on the day before *Pf* sporozoite challenge showed varying binding responses (ranging from 0.2083 to 0.8661 nm) to NPNA3 peptide indicating the differences in quantity of NPNA3 specific IgG antibodies (**Figure 5A**). The dissociation phases of these serum IgG antibodies interaction with NPNA3 were biphasic (**Figure 5A**). The standard analysis of dissociation phases yielded a median *k_d_* 1.1 × 10^-3^ s^-1^ (ranging from 0.7 to 2.2 × 10^-3^ s^-1^) as depicted in **Figure 5C**. In comparison, PAART analysis of the same dissociation courses shown in **Figure 5A** resulted in better fit of the data (**Figure 5B**) as judged by the χ^2^ values of the fits; PAART analysis median χ^2^ 2.9×10^-4^ (range 2.3×10^-4^ – 4.1×10^-4^) compared to the standard analysis median χ^2^ 2.4×10^-2^ (range 1.9×10^-3^ – 9.0×10^-2^). As shown in **Figure 5D**, the PAART analysis yielded two *k_d_* values; a slower *k_d_* (median *k_d_* = 7.3× 10^-4^ s^-1^) similar to the *k_d_* obtained by standard analysis and a faster *k_d_* (median *k_d_* = 1.0× 10^-2^ s^-1^). The fraction of slower *k_d_* was higher (79.2 to 94.8%) than the fraction of faster *k_d_* (5.2 to 20.8%). These percentages do not represent the fractions of the antibodies associated with each of the *k_d_* but rather the fractions of antigen occupancy at the beginning of dissociation. In short, for malaria vaccinees’ IgG antibodies binding to NPNA3 antigen, the PAART analysis of dissociation phases has separated the avidity of antibodies into two bins differing in median *k_d_* by roughly 14-fold, revealing the avidity diverseness of NPNA3 specific antibodies.

### PAART resolves up to three antibody avidity components in Typhoid vaccinees serum IgG antibodies binding to Vi polysaccharide antigen

**Figure 6:**
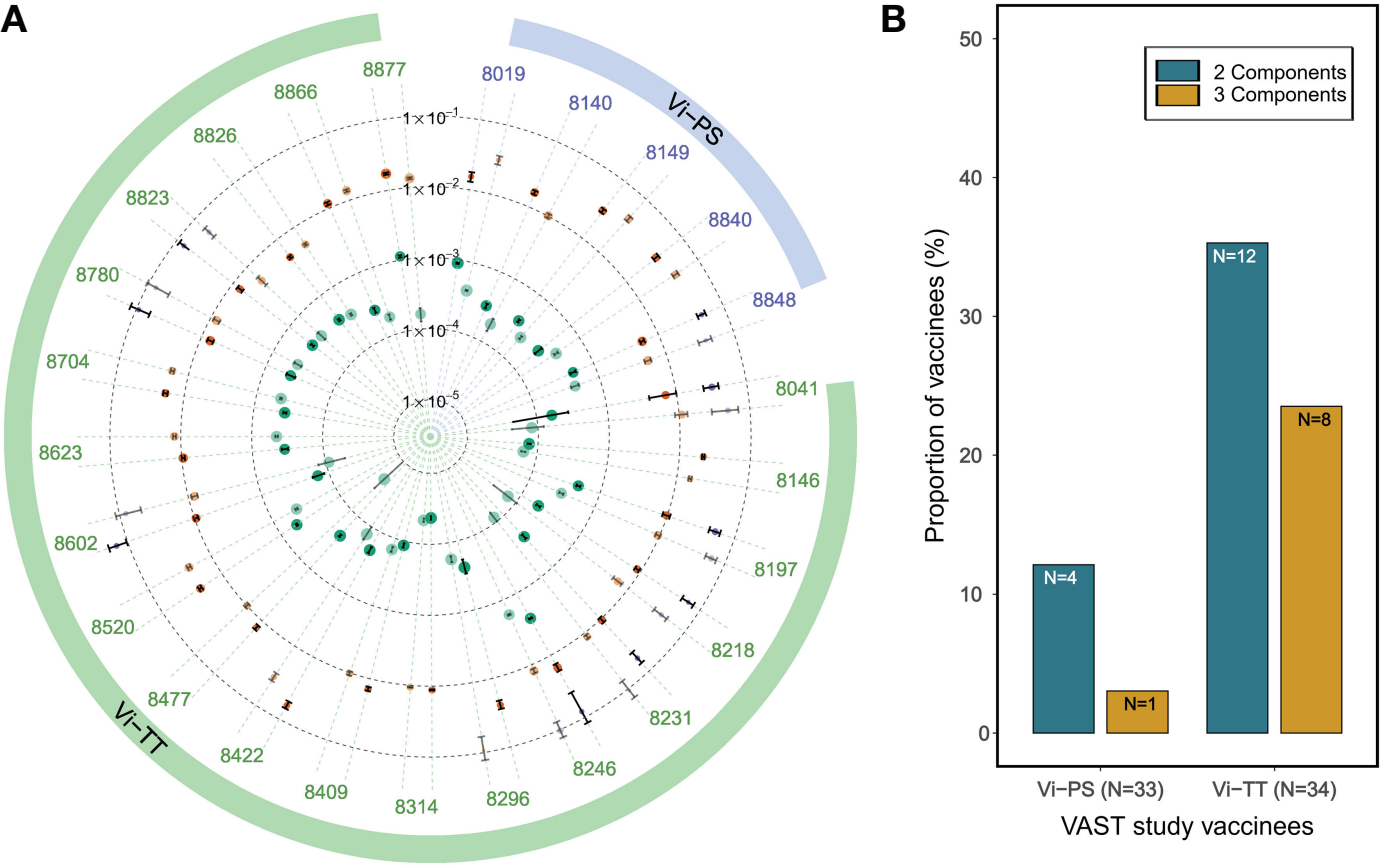
PAART analysis differentiates the avidity heterogeneity between Typhoid vaccine arms. **(A)** A radial plot of different *k_d_* values dissected from the dissociation phases (in duplicate) of serum IgG antibodies of VAST clinical trial participants that received either a Vi-PS or a Vi-TT vaccine are shown. Duplicate data shown are the best two replicates of the triplicate time-courses measured. **(B)** The proportion of vaccinees with ≥5% of 2 and 3 antibody avidity components resolved by PAART analysis are shown for Vi-PS and Vi-TT groups of VAST clinical trial participants. In panel A, the error bars associated with symbols indicate the standard error in the estimate of *k_d_* values.

As an additional testing, we applied the PAART analysis to polyclonal IgG antibodies purified from the participants’ sera of the Vaccines Against *Salmonella Typhi* (VAST) trial (29). We used purified IgG antibodies from VAST trial vaccinees that received a single dose of either a purified Vi polysaccharide (Vi-PS) or a Vi tetanus toxoid conjugate (Vi-TT) vaccine, and reported *k_d_* values of Vi-PS interaction obtained using standard analysis previously (11). Here we focused on the Day 0 time point (4 weeks after immunization) serum IgG for PAART analysis of dissociation time-courses. Interestingly, 2-3 antibody components were resolved by PAART analysis (**Figure 6**). Serum IgG antibodies 28 days post vaccination revealed a higher proportion of participants that exhibited two or more *k_d_* resolved in the Vi-TT group than in the Vi-PS group (**Figure 6B**). The slower *k_d_* (2×10^-5^ to 1×10^-3^ s^-1^) were associated with higher responses (60 to 95% of the total response) than the >1×10^-2^ s^-1^ *k_d_* (5 to 50% of the responses) as shown in **Figure 6A**. The Vi-PS being a polymeric antigen likely presents different epitopes that can be targeted by the vaccine elicited antibodies and hence the heterogeneity in avidity observed here could arise not only due to competition but also due to difference in fine specificities (33). Overall, PAART analysis dissected the avidity of polyclonal IgG antibodies of most of the VAST study vaccinees into at least two bins differing in dissociation rates (*k_d_* ranging from 2×10^-5^ to 1×10^-3^ s^-1^ and *k_d_* ≥1×10^-2^ s^-1^) and further revealed the inter-group difference in proportion of avidity diverseness.

### PAART analysis fine-resolves affinity maturation of malaria vaccine-induced serum antibody responses to different CSP antigens

**Figure 7:**
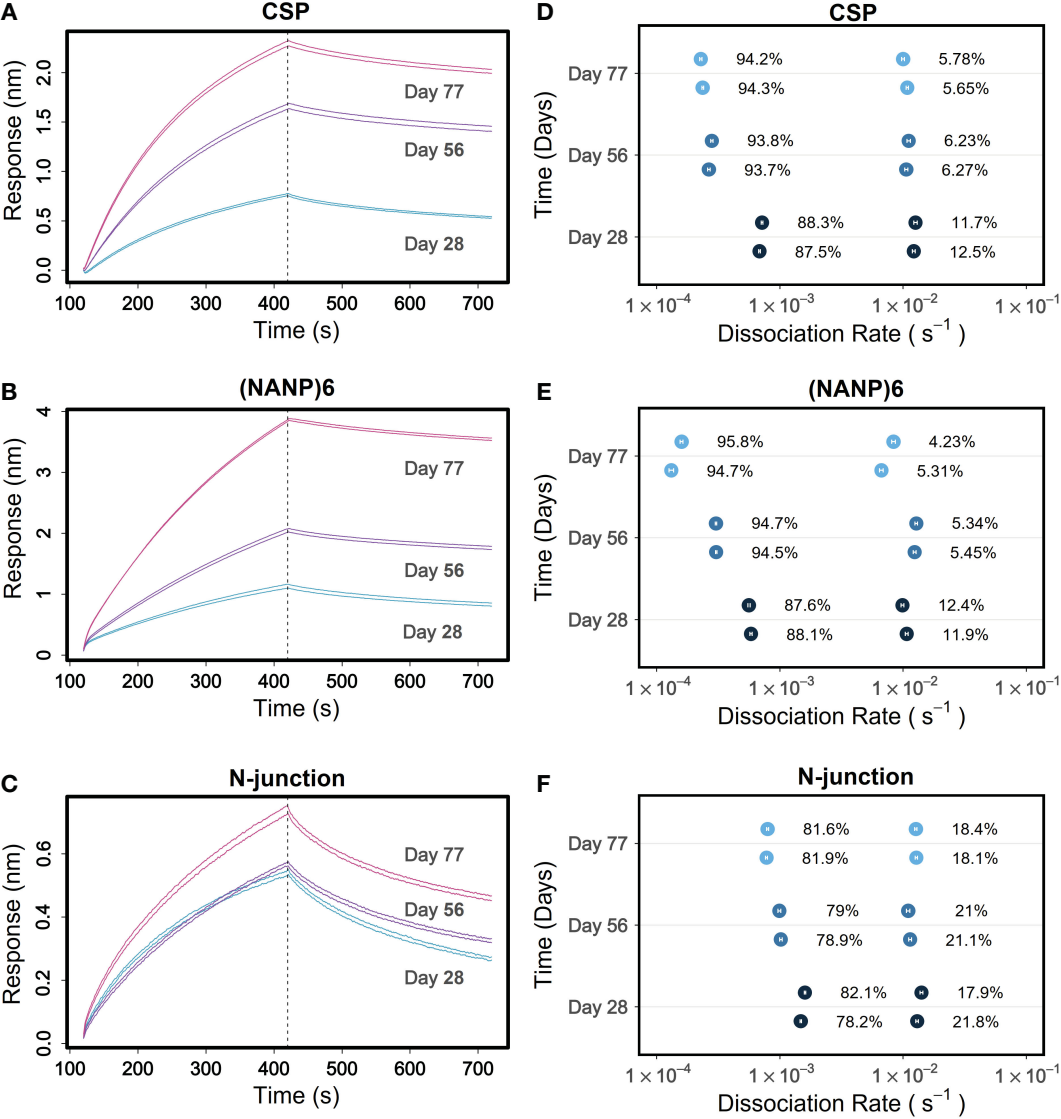
Tracking of fine-resolved affinity maturation of antibody responses to different CSP antigens elicited in a protected RTS,S/AS01 malaria vaccinee. **(A–C)** Time-courses of 1:50 diluted sera from days 28, 56 and 77 corresponding to post-immune 1, 2 and 3 time points respectively of a protected RTS,S/AS01 vaccinee are shown for binding to antigens recombinant CSP **(A)**, NANP6 peptide **(B)** and N-interface peptide **(C)** in duplicate. **(D–F)** Different *k_d_* values dissected for each replicate at days 28, 56 and 77 are shown for binding to CSP **(D)**, NANP6 **(E)** and N-interface **(F)**. In panels **(D–F)**, the error bars associated with symbols indicate the standard error in the estimate of *k_d_* values.

The kinetics method of tracking affinity maturation of polyclonal antibody responses involves monitoring the *k_d_* of the interaction of longitudinal samples with the antigens of interest. A decrease in *k_d_* values, going from the samples drawn at early time points of immunization or pathogen exposure to those drawn at later time points, would indicate affinity maturation as the slower *k_d_* values are associated with higher avidity antibodies. Since it can dissect different avidities, PAART stands in good stead to fine-resolve affinity maturation of polyclonal antibody responses towards antigens. To demonstrate this utility, we perused a longitudinal data set of a phase 2 malaria vaccine trial (NCT01366534) participants’ sera binding kinetics that we reported earlier (8, 30). PAART analysis was performed for vaccinees that showed serum binding responses higher than the limit that is optimal for dissociation rate determination for a given antigen at all post-immune time points. Data obtained on sera of a vaccinee protected against *Pf* sporozoite challenge are shown in **Figure 7** to showcase the fine resolution of affinity maturation of antibody responses. The time-courses of post-immune 1, 2 and 3 sera (Days 28, 56 and 77 respectively) of a protected vaccinee receiving the standard dose of RTS,S/AS01 interacting with CSP antigens are shown in **Figures 7A–C**. The CSP antigens tested include a recombinant CSP (**Figure 7A**), NANP6; a peptide corresponding to the central repeat region of CSP (**Figure 7B**) and N-interface; a peptide corresponding the junctional region immediately upstream of the central repeat region of CSP (**Figure 7C**). The RTS,S vaccine does not include the N-terminal junctional region, but the NANP repeat specific antibodies induced by RTS,S vaccination, as reported earlier, cross react with it (**Figure 7C**). The antigen specific binding responses were low at Day 28 and increased at Days 56 and 77 showing increase in antibody magnitude after second and third immunizations (**Figures 7A–C**). PAART analysis derived two different *k_d_* values for CSP and NANP6 binding at Day 28; one with a *k_d_* in the order of 10^-4^ s^-1^ (average *k_d_* 7.0×10^-4^ s^-1^ and 5.7×10^-4^ s^-1^ for CSP and NANP6 respectively) and another with a *k_d_* in the order of 10^-2^ s^-1^ (average *k_d_* 1.2×10^-2^ s^-1^ and 1×10^-2^ s^-1^ for CSP and NANP6 respectively) corresponding to ~88 and 12% respectively of the total binding responses (**Figures 7D, E**). Interestingly, at Day 56 the slower *k_d_* values decreased further (to 2.7×10^-4^ and 3.1×10^-4^ s^-1^ for CSP and NANP6 respectively) contributing to ~94% of total binding response whereas the faster *k_d_* values remained essentially unchanged but with a decreased contribution (5%) to the total binding response (**Figures 7D, E**). At Day 77, a < 2-fold decrease in slower *k_d_* values was noted for CSP and NANP6 binding with an essentially unchanged faster *k_d_* values and percent contribution to total binding of both faster and slower *k_d_* values. These results indicate that in this protected vaccinee, there remains a population of NANP6- and CSP specific antibodies heterogeneous in avidity after the first RTS,S/AS01 immunization; a stronger one that matures 4-fold in avidity upon second and third immunization to contribute predominantly to the observed binding plus a weaker one that did not affinity mature yet. PAART analysis resolved two different *k_d_* values (average values 1.5×10^-3^ s^-1^ and 1.4×10^-2^ s^-1^) from the N-interface binding of protected vaccinee’s serum at Day 28 as well (**Figure 7F**). Unlike the 4-fold decrease in the slower component (*k_d_* values in the order of 10^-4^ s^-1^) observed for NANP6 and CSP binding, there was only a marginal decrease in *k_d_* (1.9 fold decrease between Day 28 Day 77) of N-interface specific slower component. Overall, these results demonstrate the utility of obtaining a fine-resolution feature of antigen specific affinity maturation of vaccine-induced serum antibody responses.

### PAART analysis resolves differences in avidity heterogeneity between protected and not-protected malaria vaccinees in a phase 2 clinical trial

**Figure 8:**
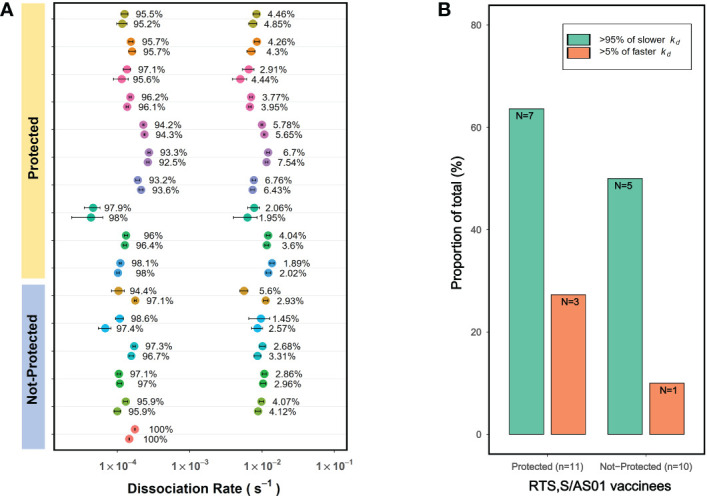
PAART analysis resolves differences in avidity heterogeneity between protected and infected malaria vaccinees in a phase 2 clinical trial. **(A)** The *k_d_* values derived from PAART analysis of dissociation time-courses of 1:50 diluted sera from visit 20 corresponding to post-immune 3 (Day of Challenge) time point are shown for standard dose regimen RTS,S/AS01 vaccinees. Two best replicates data from PAART analysis are shown. **(B)** The proportion of total vaccinees from the protected and not-protected groups with >95% of slower *k_d_* and >5% of faster *k_d_* are shown. Binding responses were below the dissociation rate quantifiable limit for 1 and 4 vaccinees from the protected and not-protected groups respectively. In panel A, the error bars associated with symbols indicate the standard error in the estimate of *k_d_* values.

Examining vaccine induced antibody avidity differences between protected and not-protected vaccinees is important in immune correlate analysis of vaccines as it would reveal whether or not the vaccine elicited antibody avidity associates with protection. Here we explored whether PAART could be applied to identify differences in heterogeneity in vaccine induced antibody avidity between protected and not-protected vaccinees. For this purpose, we used the dissociation kinetics data obtained for a phase 2 malaria vaccine trial specimen reported earlier (8, 30). In the dissociation rate measurement analysis we reported earlier (8), on the day of challenge (visit 20, post-dose 3), vaccinees receiving the RTS,S/AS01 standard dose showed no significant difference in CSP-specific serum antibody avidity (*k_d_*) between the protected and not-protected vaccinees from Pf sporozoite infection. Interestingly, PAART analysis showed that all but one vaccinee serum had two antibody components; one contributing dominantly to the total binding response (93-98%) with mean *k_d_* ~ 1×10^-4^ s^-1^ and another contributing only 2-7% to the total binding response with a *k_d_* ~ 1×10^-2^ s^-1^ (**Figure 8A**). When compared, 36.4% (4 out of 11) of the protected vaccinees exhibited >5% of the weak avidity antibody component whereas only 10% (1 out of 10) of the not-protected vaccinees had more >5% of the weak avidity antibody component (**Figure 8B**). Since CSP contains various epitopes, the epitope specificities of the two antibody components cannot be assigned. It would require further probing to understand why more protected subjects have weak avidity antibodies. In brief, these results demonstrate the capability of PAART to probe the differences in heterogeneity of avidity between protected and not-protected vaccinees in clinical trials.

### Polyclonal Fab provide enhanced resolution compared to polyclonal IgG antibodies in identifying antibody components

**Figure 9:**
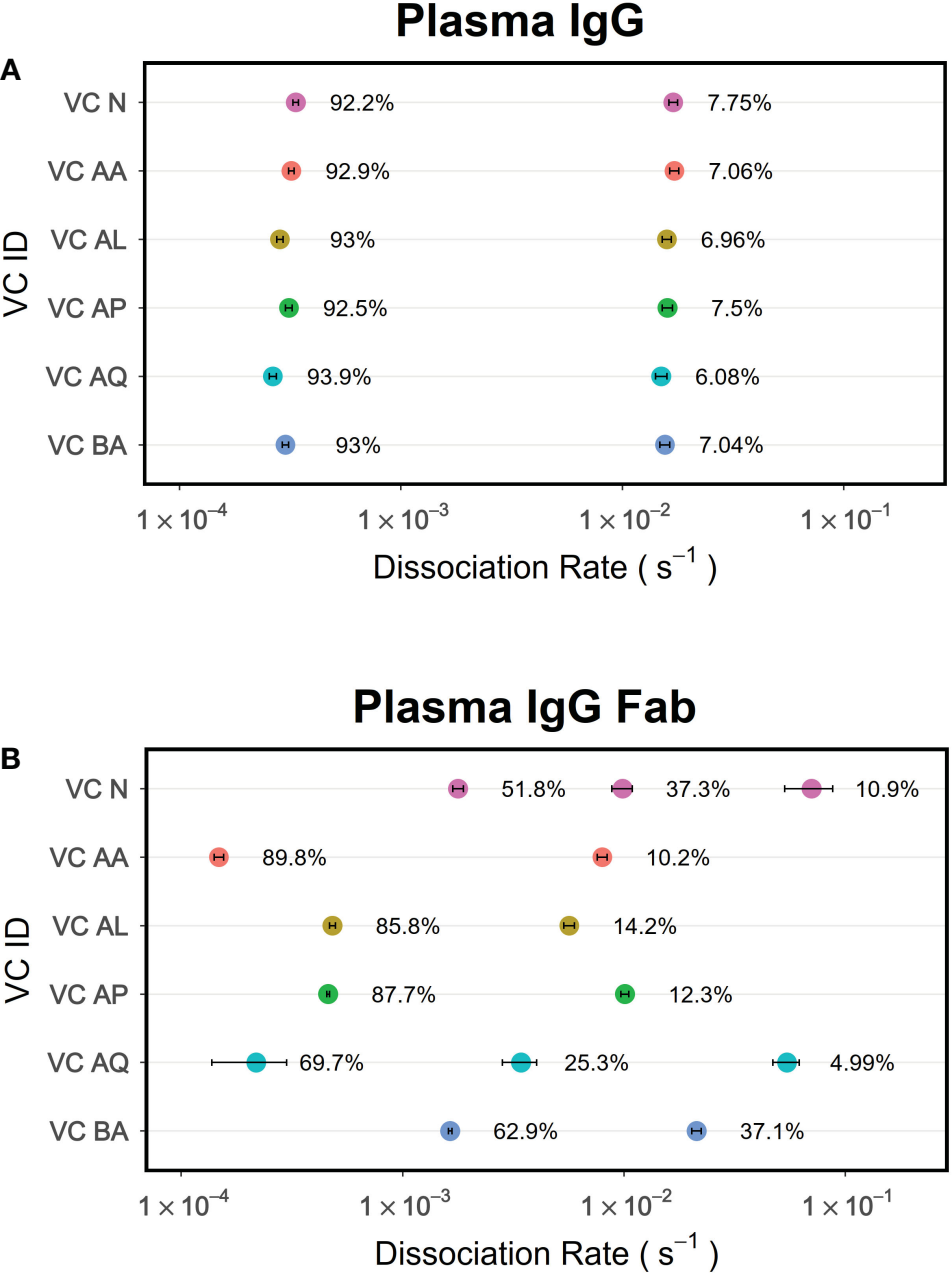
PAART analysis of polyclonal antibody Fab dissociation rather than the polyclonal IgG antibodies provides enhanced resolution of antibody heterogeneity. Data of polyclonal IgG antibodies **(A)** and the corresponding Fab **(B)** from the HIV-1 virus controllers interacting with BG505gp140 T332N SOSIP.664 antigen were analyzed using PAART to derive the minimal number of *k_d_* values needed to explain the dissociation phase. The error bars associated with symbols indicate the standard error in the estimate of *k_d_* values.

While immobilization of an antigen on sensor surfaces facilitates developing an antibody-antigen binding assays that are convenient for probing serum and plasma samples, it also improves the bivalent interaction of antibodies due to higher local concentration of antigen. Thus, the binding results obtained in this format includes an avidity effect (34, 35). If the antigen is not monomeric, the avidity effect will be compounding. One way to minimize the avidity effect and measure affinity is to immobilize antigens at low density so that antibody-antigen interactions are monovalent. An alternate strategy would be to use antibody Fab instead of intact antibodies. The former strategy might not be possible when working with serum or plasma samples as low abundance antibodies binding would not be detected. Therefore, using antibody Fab is a preferred way for measuring average affinity of pAbs. Here we demonstrate that the dissociation phase data obtained for polyclonal Fab when analyzed by PAART can provide enhanced resolution in dissecting different antibody components as compared to using data obtained from the corresponding IgG antibodies. Nyanhete et al. (22) recently reported broadly HIV-1 neutralizing polyclonal antibody activity in a subset of virus controllers (VCs). We performed PAART analysis on the polyclonal antibody Fab and the intact IgG antibodies from VCs plasma binding to a native like HIV-1 envelope glycoprotein BG505gp140 T332N SOSIP.664. The *k_d_* values from the PAART analysis of the VCs plasma IgG and Fab dissociation from BG505gp140 T332N SOSIP.664 are shown for **Figures 9A, B** respectively. Two *k_d_* values were resolved for the dissociation of polyclonal plasma IgG of all six virus controllers; one corresponding to higher avidity (*k_d_* ranging from 2.6 – 3.4×10^-4^ s^-1^) with a dominant contribution (92.2 – 93.9%) to the binding response and another attributable to lower avidity (*k_d_* ranging from 1.5 to 1.7×10^-2^ s^-1^) with a minor contribution (6.1 – 7.8%) to the binding. On the other hand, the polyclonal antibody Fab dissociation of VCs were resolved into 2 to 3 antibody components with different *k_d_* values (**Figure 9B**). Unlike the IgG dissociation data that did not vary between VCs either in the slower or the faster *k_d_* values, the PAART derived *k_d_* values of the Fab dissociation data showed a marked difference between VC (**Figure 9B**). When compared with the slower *k_d_* values of IgG data (**Figure 9A**), the slower *k_d_* values resolved for Fab dissociation were similar in two VC (VC AA and VC AQ), 2 fold faster in two VC (VC AL and VA AP) and ~ 10 fold faster in two VC (VC N and VC BA) pointing out the differing levels of the affinity of the pAbs that contributed to the total binding of BG505gp140 T332N SOSIP.664 (**Figure 9B**). The VCs exhibited higher fractions of faster *k_d_* values for Fab dissociation than the IgG dissociation. Differences in faster *k_d_* values between VCs were also observed and a third *k_d_* was also resolved in VC N and VC AQ Fab dissociation. Overall, these results exemplify the utility of PAART and polyclonal Fab-antigen binding kinetics data to get enhanced resolution of antibody heterogeneity.

## Discussion

Characterization of pAbs for the distribution of specificity (22, 36, 37) and avidity (38, 39) by different techniques has been reported recently. Our focus is on utilizing pAbs-antigen binding kinetics data obtained with the commonly employed label-free platforms to dissect distributions of avidities. The pAbs-antigen interaction time-courses comprise important kinetics information that can reveal the underlying antibody dynamics. Avidity distributions need to be mined out from the time-courses of pAbs-antigen interaction for better understanding the antibody dynamics that are occurring during this interaction. While delineation of antibody avidity heterogeneity down to clonal level is not feasible, binning the pAbs into various groups differing in avidity is achievable by appropriate modeling of the dissociation phases. To accomplish this, we have reported here the development, validation and application of PAART for analyzing the dissociation phase of pAbs-antigen interaction data. This is a substantial development in enabling avidity binning of pAbs which yields additional insights in the antibody dynamics compared to the weighted avidity obtained using standard analysis fitting of the antibodies dissociation time-courses to a 1:1 Langmuir dissociation model.

The sum of exponentials model utilized in the PAART has been employed previously in fragment-based drug discovery using dissociation phase screening of crude reaction mixtures that contain low affinity starting material and different amounts of desired high affinity product(s) for target ligand (40). In fragment-based discovery, this has been restricted to a system of only two components. In contrast, PAART has been designed to resolve more than two dissociation rate components and is equipped to guard against overfitting of data to select a parsimonious model for a given dissociation course using Akaike information criterion. PAART has been successfully tested using binary mixtures of mAbs differing in dissociation rates to recover appropriate fractions of different dissociation rates accounting for the antibody competition.

Application of PAART to malaria and typhoid vaccinees’ IgG antibodies-antigen binding data revealed heterogeneity in the avidity and identified inter-group differences in avidity heterogeneity of typhoid vaccinees’ antibodies. The additional information of avidity heterogeneity could provide more insights when correlated with the vaccine efficacy. Additional application of PAART includes tracking of component level affinity maturation of pAbs over time in response to vaccination or exposure to pathogens. As an example, we demonstrated tracking the fine-resolved affinity maturation of antibody response against different CSP antigens in a protected malaria vaccinee (**Figure 7**); an affinity maturing antibody component and the other that did not mature. The latter could be due to lack of affinity maturation of antibodies elicited after first immunization or due to the emergence of new antibody responses after second and third immunization that are yet to affinity mature. Affinity maturation tracking *via* dissociation rate binning could be used to make comparisons between individuals, groups or vaccine candidates to reveal fine-differences that would help form strategies intended to guide the host immune response in a desired fashion. PAART could also be applied to better understand vaccine breakthrough infections by analyzing the differences in avidity heterogeneity of vaccinees’ antibodies to the vaccine antigen and antigens representing the evolving variants.

Successful utility of PAART in the analysis of pAbs will depend on the choice of antigen and assay conditions. The first choice should be the use of antigen constructs that present a minimal epitope of interest such as the ones targeted by protective/neutralizing antibody responses, compared to a full-length antigen. It will be advantageous as the binned avidity of antibodies studied will be specific to the epitope. The epitope-specific dissociation rate binning data can be used to draw a correlation with the functional activity of the polyclonal sample. It may be easier to pursue this strategy for linear epitopes but might require design and production of antigen constructs for presenting conformational epitopes. If full-length antigens or multi-epitope antigens are used, the fine specificity of binned dissociation rates will remain unknown and might render it difficult to draw correlation with functional data. If dissociation rate binning can be done using full-length antigen and as many minimum epitopes/domains as needed, comprehensive dissociation rate binning database could be built for drawing correlation with different functional properties. Regarding assay conditions, as outlined in our testing, the length of association phase will determine the antigen occupancy of antibodies if competition is involved. Therefore, a lengthy association time e.g 10 minutes or more should be avoided so that detection of weak avidity antibodies does not get lost. Similarly, shortening the association phase will decrease antigen occupancy by antibodies with slow association rate. Thus, 2 to 5 minutes monitoring of association phase before following dissociation phase would be appropriate. Another point to note is that choosing an appropriate window of the dissociation phase is important for a meaningful analysis. Distortion in the binding response signal is not uncommon during the initial few seconds of the dissociation time-courses and should be excluded in the analysis as done in Langmuir dissociation analysis. Similarly, residual non-specific binding, if any, towards the end of the dissociation phase should be excluded from the analysis window to avoid PAART resolving that phase as a slow dissociation rate contributing to the overall dissociation. An alternative option is to include a term corresponding to the response at infinite time (R_∞_) in the sum of exponentials model employed by PAART.

Limitations of PAART include the decreased resolving power if the difference in *k_d_* values of antibodies in a sample is small. Another limitation is to assign epitope specificity of the dissociation rate binned antibodies if the interaction followed was with a multi-epitope antigen. However, together with additional investigations using other techniques such as electron microscopy polyclonal epitope mapping (22), PAART would be valuable in understanding the distributions of specificity and avidity of pAbs.

To conclude, we have developed and demonstrated the capability of an analytical tool for dissociation rate binning of pAbs-antigen interaction time-courses. The binned dissociation rates reveal the heterogeneity in the avidity of pAbs and the fractions of binding response associated with these different dissociation rates indicate the respective antigen occupancy levels of the binned antibodies. The dissociation rate binning data obtained using PAART analysis could be applied in immunogenicity analyses, evaluating vaccine constructs, vaccine formulations and also for tracking affinity maturation.

## Data availability statement

The raw data supporting the conclusions of this article will be made available by the authors, without undue reservation.

## Ethics statement

The studies involving human participants were reviewed and approved by Walter Reed Army Institute of Research (WRAIR) Institutional Review Board and the PATH-Malaria Vaccine Initiative’s Western Institutional Review Board for study NCT01366534; United Kingdom National Research Ethics Service, Committee South Central–Oxford A (reference 13/SC/0208), the Western Institution Review Board (reference 20130698), and the United Kingdom Medicines and healthcare Products Regulatory Agency (reference 21584/0317/001-0001) for study NCT01883609; South Central Oxford A Ethics Committee (reference 14/SC/1427) for study NCT02324751; and Duke University Institutional Review Board for HIV-1 virus controller cohort. The patients/participants provided their written informed consent to participate in this study.

## Author contributions

MD, SMD, and GDT conceived and designed study. MD developed code, RLS and KL performed code optimization. UW-R, EJ, KJE, AVSH, CJ, JH, and AJP provided study samples. SD made reagent. MA, RHCH, LCD, and TN performed experiments, KL and RLS analyzed data. SMD wrote the manuscript. KL, MD, RLS, LCD, UW-R, EJ, SMA, and GDT edited manuscript. MA, RHCH, TN, SD, KJE, AVSH, CJ, JH, and AJP reviewed manuscript. All authors contributed to the article and approved the submitted version.

## Footnotes

1 Li, K., H.C. Huntwork, R., Dodds, M., L. Spreng, R., & Dennison, S. M. Polyclonal Antibodies Avidity Resolution Tool (PAART) [Computer software] https://github.com/DukeCHSI/PAART.
