# Supplementary material for: A tool for evaluating heterogeneity in avidity of polyclonal antibodies
Source: Front Immunol. 2023 Feb 16;14:1049673. doi: 10.3389/fimmu.2023.1049673 (PMC9978818; doi:10.3389/fimmu.2023.1049673)
Supplement: Supplementary file 1 [file DataSheet_1.pdf]

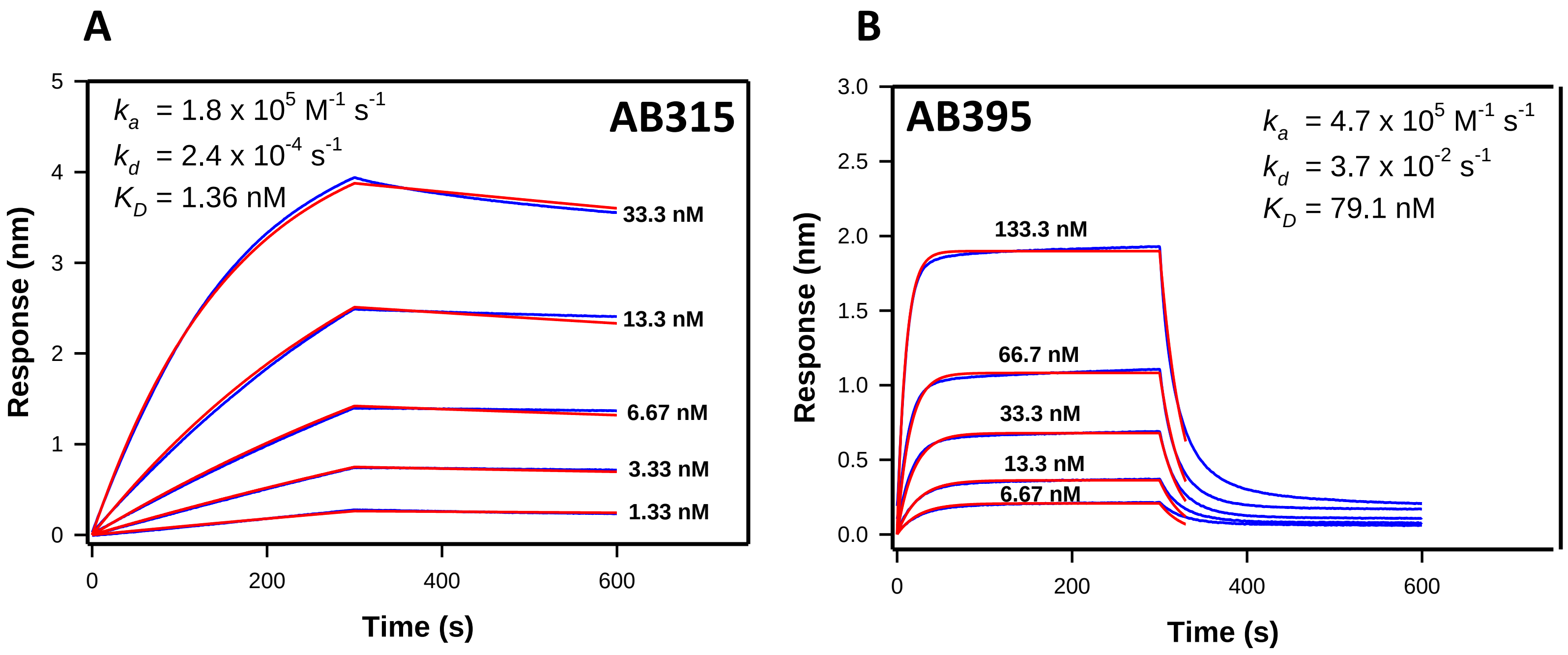

Figure S1: Binding kinetics of anti-CSP mAbs differing in avidity by Biolayer Interferometry. The specific binding time courses (blue lines) of central repeat region targeting anti-CSP mAbs AB315 (A) and AB395 (B) binding to a NANP repeat peptide NPNA3 at indicated concentrations are shown along with the best fits (red lines) to a 1:1 binding model.
