# Supplementary material for: A tool for evaluating heterogeneity in avidity of polyclonal antibodies
Source: Front Immunol. 2023 Feb 16;14:1049673. doi: 10.3389/fimmu.2023.1049673 (PMC9978818; doi:10.3389/fimmu.2023.1049673)
Supplement: Supplementary file 2 [file DataSheet_2.pdf]

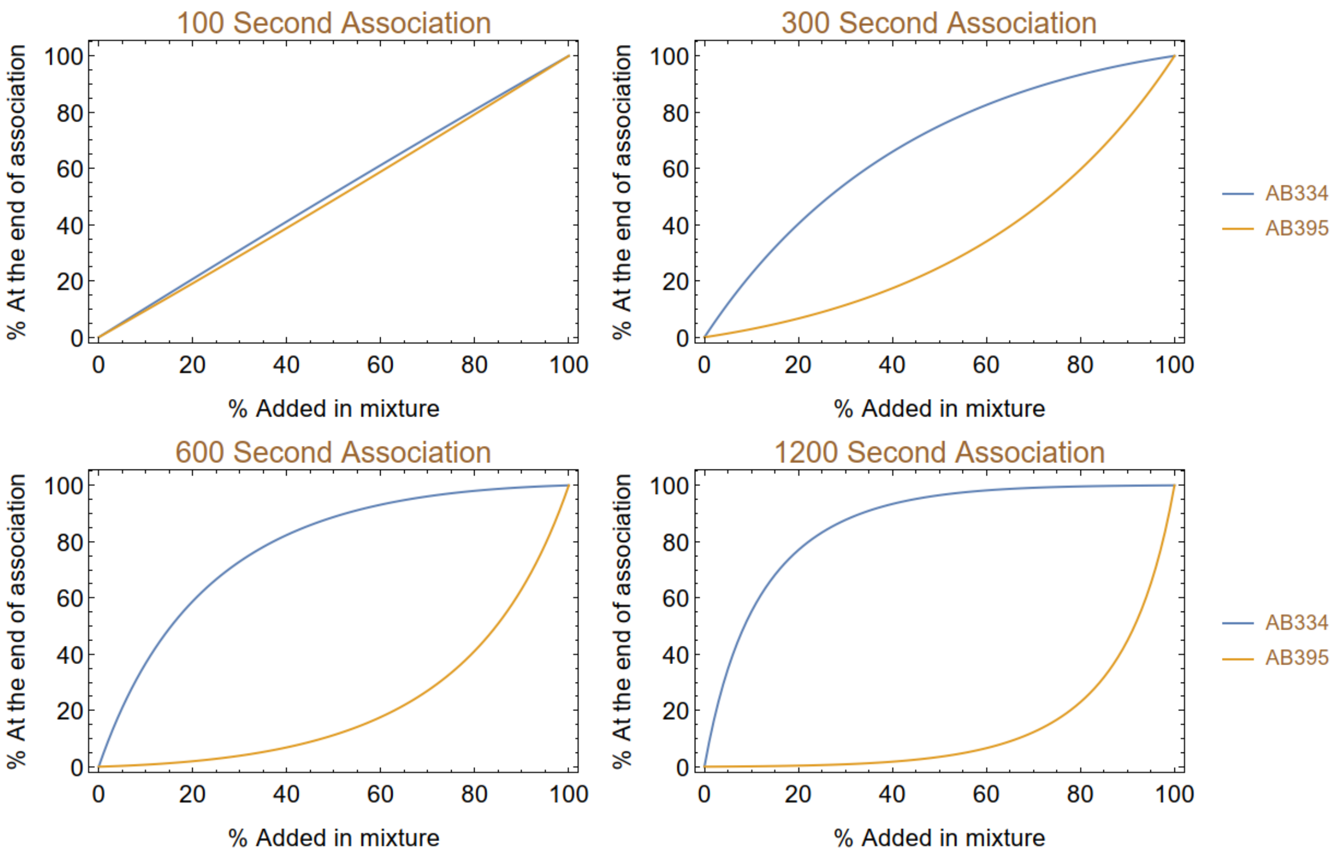

Figure S2: Antigen occupancy of component antibodies of binary mixtures of mAbs AB334 and AB395 is dependent on the duration of association step. The fractions of NPNA3 antigen binding response of high avidity mAb AB334 and low avidity mAb AB395 in binary mixtures at different compositions are shown for different lengths of association step. The fractions (in %) shown for different compositions at different lengths of association step were calculated from simulated binding responses of component antibodies at different association times indicated. Simulations of competing antibodies AB334 and AB395 binding to the NPNA3 ligand were performed using linear solutions obtained by solving differential equations $\frac{dR_{AB334}}{dt} = k_{a\ AB334} \times C_{AB334} \times (R_{max} - R_{AB334} - R_{AB395}) - k_{d\ AB334} \times R_{AB334}$ and $\frac{dR_{AB395}}{dt} = k_{a\ AB395} \times C_{AB395} \times (R_{max} - R_{AB334} - R_{AB395}) - k_{d\ AB395} \times R_{AB395}$. Here, R_{AB334} and R_{AB395} are responses contributed to total binding by mAbs AB334 and 395 respectively, R_{max} is the theoretical maximum binding response, $k_{a\ AB334}$ and $k_{a\ AB395}$ are the association rate constants of AB334 and AB395 respectively, $k_{d\ AB334}$ and $k_{d\ AB395}$ are dissociation rate constants of AB334 and AB395 respectively and C_{AB334} and C_{AB395} are the concentrations of AB334 and AB395 respectively in the binary mixtures. Mathematica 12.0 (Wolfram, Champaign, IL) program's 'Dsolve' function was used to solve these differential equations and run the simulations with the inputs of k_a and k_d values of the mAbs from Table 1 and the concentrations of antibodies in the binary mixtures of different compositions.
