# Supplementary material for: A tool for evaluating heterogeneity in avidity of polyclonal antibodies
Source: Front Immunol. 2023 Feb 16;14:1049673. doi: 10.3389/fimmu.2023.1049673 (PMC9978818; doi:10.3389/fimmu.2023.1049673)
Supplement: Supplementary file 3 [file DataSheet_3.pdf]

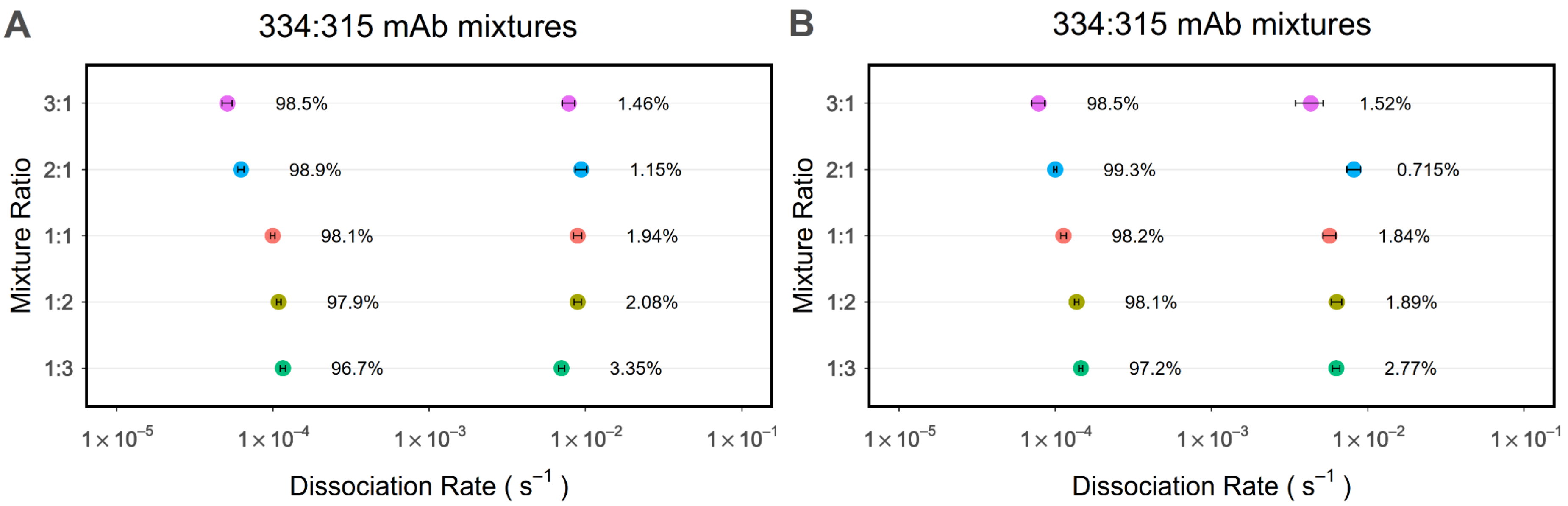

Figure S3: Dissociation rates dissected from PAART analysis of dissociation phases of binary mixtures of two high avidity monoclonal antibodies. The dissociation rates and their fractions estimated by PAART analysis of dissociation phases of the binary mixtures of AB334 and AB315 at various compositions are shown. The dissociation phases used here were recorded after 5 minutes (A) and 30 minutes (B) of association phase.

.
